# Modeling of shotgun sequencing of DNA plasmids using experimental and theoretical approaches

**DOI:** 10.1186/s12859-020-3461-6

**Published:** 2020-04-03

**Authors:** Sergey Shityakov, Elena Bencurova, Carola Förster, Thomas Dandekar

**Affiliations:** 1 Department of Bioinformatics, University of Würzburg, 97074 Würzburg, Germany; 2 Department of Psychiatry, China Medical University Hospital, 40402 Taichung, Taiwan; 3 Department of Anesthesia and Critical Care, Würzburg University Hospital, 97080 Würzburg, Germany

**Keywords:** Shotgun method, Sanger sequencing, Virtual sequencing, Polymerase chain reaction, Gene expression vectors, Synthetic biology

## Abstract

**Background:**

Processing and analysis of DNA sequences obtained from next-generation sequencing (NGS) face some difficulties in terms of the correct prediction of DNA sequencing outcomes without the implementation of bioinformatics approaches. However, algorithms based on NGS perform inefficiently due to the generation of long DNA fragments, the difficulty of assembling them and the complexity of the used genomes. On the other hand, the Sanger DNA sequencing method is still considered to be the most reliable; it is a reliable choice for virtual modeling to build all possible consensus sequences from smaller DNA fragments.

**Results:**

In silico and in vitro experiments were conducted: (1) to implement and test our novel sequencing algorithm, using the standard cloning vectors of different length and (2) to validate experimentally virtual shotgun sequencing using the PCR technique with the number of cycles from 1 to 9 for each reaction.

**Conclusions:**

We applied a novel algorithm based on Sanger methodology to correctly predict and emphasize the performance of DNA sequencing techniques as well as in de novo DNA sequencing and its further application in synthetic biology. We demonstrate the statistical significance of our results.

**Graphical abstract:**

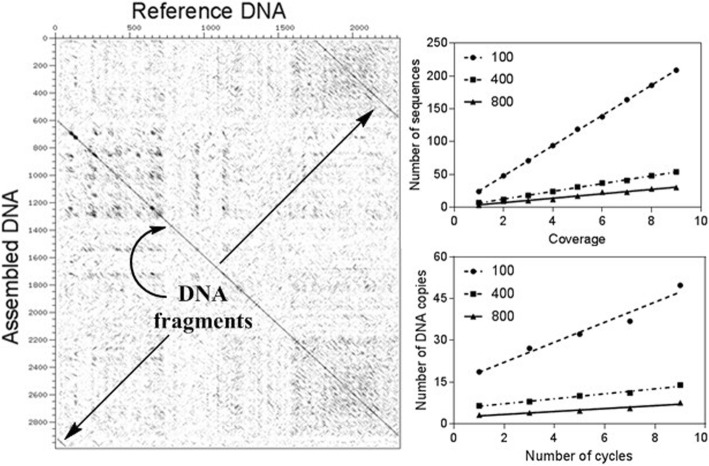

## Background

Optimization in the processing of DNA sequence data may impose a serious challenge regarding the correct prediction of DNA sequencing outcomes without the application of bioinformatics approaches [1]. These approaches play an important role in novel sequencing pipelines, termed Next-Generation Sequencing (NGS) technologies, and they have transformed the sequencing landscape in the past few years [2, 3]. Despite significant scientific achievements in DNA sequencing, there is still a shortage of efficient bioinformatics tools for virtual NGS simulations due to the generation of long DNA fragments and difficulty assembling them [4, 5]. Although some computational algorithms have already been developed and tested for the construction of a realistic data set, such as the MetaSim simulator to model Roche’s 454 and Illumina technologies, they still lack thorough experimental validation of the generated results [6]. Moreover, these algorithms deal with NGS, which might be error-prone [7]. Thus, they may lead to artificial mutations and sequencing bias [7]. In particular, a study of NGS biases defined that introns are less covered with reads than exons due to the much higher complexity of the latter structures [8].

On the other hand, the Sanger method is a termination sequencing technology for determining a nucleotide sequence of DNA molecules that can only be used for short DNA strands of 100 to 1000 base pairs [9], which is suitable for the sequencing of small DNA plasmids. For example, Sentchilo et al. used both Sanger sequencing and 454- sequencing combined with classic CsCl density gradient centrifugation, to characterize a wastewater metagenome of plasmids to determine some larger circular genetic elements [10]. The conventional Sanger method is still considered the “gold standard,” it is the most reliable sequencing methodology, but it might be also laborious and time-consuming [11, 12].

Some attempts have been made to develop open-source bioinformatics tools, simulating shotgun (genomic, metagenomic, transcriptomic, and metatranscriptomic) datasets from reference sequencing platforms, such as the Grinder and Tracembler programs [13, 14]. However, the virtual shotgun sequencing mimicking the Sanger method of the standard cloning vectors with different length sizes has yet to be tested and validated experimentally, using polymerase chain reaction. Therefore, we implemented the virtual sequencing algorithm based on the Sanger methodology to correctly predict and emphasize the performance of this DNA sequencing technique, using the average sequence length for the adjustment of coverage values in experimental settings.

### Implementation

#### Plasmid selection

The sequencing data for the pCR™4-TOPO® plasmid containing 125 bp insertion (Thermo Scientific, Germany), pQE-30-UA-mCHERRY-GFP (in-house modified vector pQE-30 UA, Qiagen, USA) and pLEXSY-Ig-1 vector for in vitro translation of Ig-like C2-type 1 protein (Jena Biosciences, Germany, [15]) were obtained in a FASTA format from our previous sequencing experiments.

#### In silico sequencing and fragment assembly

The Sequencer algorithm developed by Bernhard Haubold (Max Planck Institute for Evolutionary Biology) was used to simulate the in silico shotgun sequencing technique for determining the nucleotide sequence of DNA molecules that are no more than a few kilobases (http://guanine.evolbio.mpg.de/sequencer). The TIGR (The Institute for Genomic Research) Assembler, a classic open-access assembly tool developed by the Institute for Genomic Research [16], was applied to build all possible consensus sequences (contigs) from smaller sequence fragments, coming from the virtual shotgun sequencing. The Dotter software, a graphical dot plot program [17], was utilized to provide the complete and detailed comparison of two analyzed sequences and to calculate the Karlin-Altschul statistics [18]. For this, the program has the ability to adjust the stringency cutoffs interactively so that the dot-matrix only needs to be calculated once [17]. The CLUSTALW 2.1 program was used for the DNA sequence alignment [19]. The DNA analysis was performed by using the BioEdit 7.0.5.3 software to calculate guanine-cytosine (GC) and adenine-thymine (AT) contents together with identity matrices between the analyzed sequences in the alignment [20].

#### PCR validation

Primers (Table 1), targeting 100, 400 and 800 bp regions of the analyzed plasmids, were designed by the Geneious PRO software (Biomatters, New Zealand). The gene-expression vectors were used as templates for PCR, containing Thermo Scientific DreamTaq Green PCR Master Mix (2x), 0.1 μM of each forward and reverse primers and 10 ng of template DNA. All reactions were performed according to the manufacturer’s instructions, with the number of cycles 1, 3, 5, 7, and 9 for each reaction. The amplicons were purified using the NucleoSpin Gel and PCR clean-up kits (Macherey-Nagel, Germany) and quantified by using the Infinite 200 PRO plate reader (Infinite® 200 PRO NanoQuant, Tecan, Switzerland). Statistical analyses were performed using linear and nonlinear regression modeling by the GraphPad Prism v.7 software for Windows (GraphPad Software, San Diego, CA). The differences were considered statistically significant at a *p*-value of < 0.05. All the necessary files (tested in Linux environment) required for the virtual sequencing, including executable programs, bash scripts, FASTA format files, and program outputs can be found in Additional file 1. All in silico experiments were performed at least three times.

**Table 1:** List of primers used in PCR

Vector	Size (nt)	Forward	Reverse
TOPO^*^	100	AATGCAGCTGGCACGACAG	AGGCACCCCAGGCTTTACA
TOPO	400	CAGCTGTGCTCGACGTTGT	GGATTCATCGACTGTGGCCG
TOPO	800	GCAGCAGATTACGCGCAGA	AATGGGCTGACCGCTTCCT
QE^**^	100	ACCGCCAAGCTGAAGGTGA	CAAGGCCTACGTGAAGCACC
QE	400	AGGTCGTTCGCTCCAAGCT	TCTACGGGGTCTGACGCTCA
QE	800	GCAGCAGATTACGCGCAGA	TAGTGTATGCGGCGACCGA
LEXSY^***^	100	TGTCTCATGAGCGGATACA	GTCTCATGAGCGGATACAT
LEXSY	400	GTCTCATGAGCGGATACAT	AGTTCGTCTTTCATCCAGTT
LEXSY	800	CTGGCGCCTCTCTAGACACA	CCGACAAGCAGAAGAACGGC

^*^-pCR™4-TOPO®; ^**^- pQE-30-UA-mCHERRY-GFP; ^***^- pLEXSY-Ig-1

## Results

**Fig. 1:**
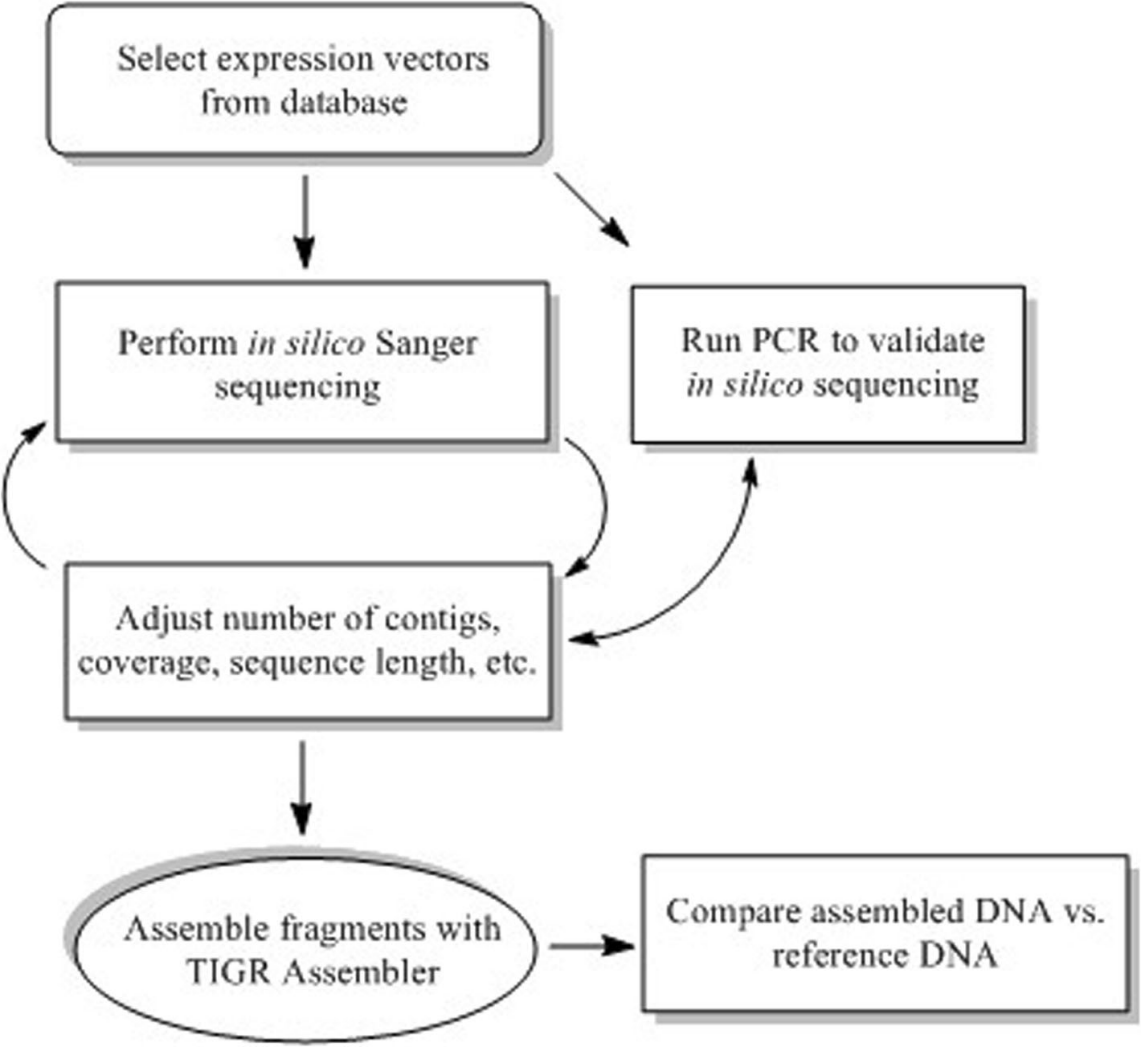
Flowchart describing the methodology used to model shotgun sequencing of DNA plasmids with experimental and theoretical approaches

The simulation of the sequencing process was used in order to optimize the DNA sequencing output for sequence assembly (Fig. 1). According to this, the resulting DNA fragments were assembled into the sought template sequence using the TIGR Assembler computer program [16]. To test this program’s performance, it is useful to simulate random DNA fragments in association with the Sequencer algorithm. In particular, the algorithm takes a template DNA sequence as input and outputs random reads until the number of sequenced nucleotides, divided by the length of the template molecule, has reached a threshold known as the coverage value.

For this purpose, we simulated this sequencing process of pCR™4-TOPO® (4079 bp), pQE-30-UA-mCHERRY-GFP (4886 bp) and pLEXSY-Ig-1 (2319 bp) cloning vectors via the Sequencer algorithm using 100, 400, and 800 bp lengths of the sequences to determine the coverage rate. In the past, most of the gene expression plasmids were being sequenced using Sanger sequencing of ~ 3 kb clone libraries, but presently it has been switched to the Roche/454 platform with GS FLX Titanium sequencing chemistry and Illumina sequencing technology [21, 22]. Therefore, the plasmid sequencing can be done by NGS, allowing us to analyze samples in a high-throughput manner with small reads of approximately 200–500 bp, but this also might be expensive and time-consuming [23]. The coverage parameter was tested in the range of 1 to 9, representing the rate at which every nucleotide in DNA should be sequenced on average. In fact, the Sanger sequencing delivers reads of up to 800 bp; however, the critical limitation is the volume of the analyzed sample and its low scalability in comparison to modern techniques [24]. Therefore, the maximal average length of the sequences was chosen to be 800 nt, which is a realistic assumption for the Sanger sequencing [25].

**Fig. 2:**
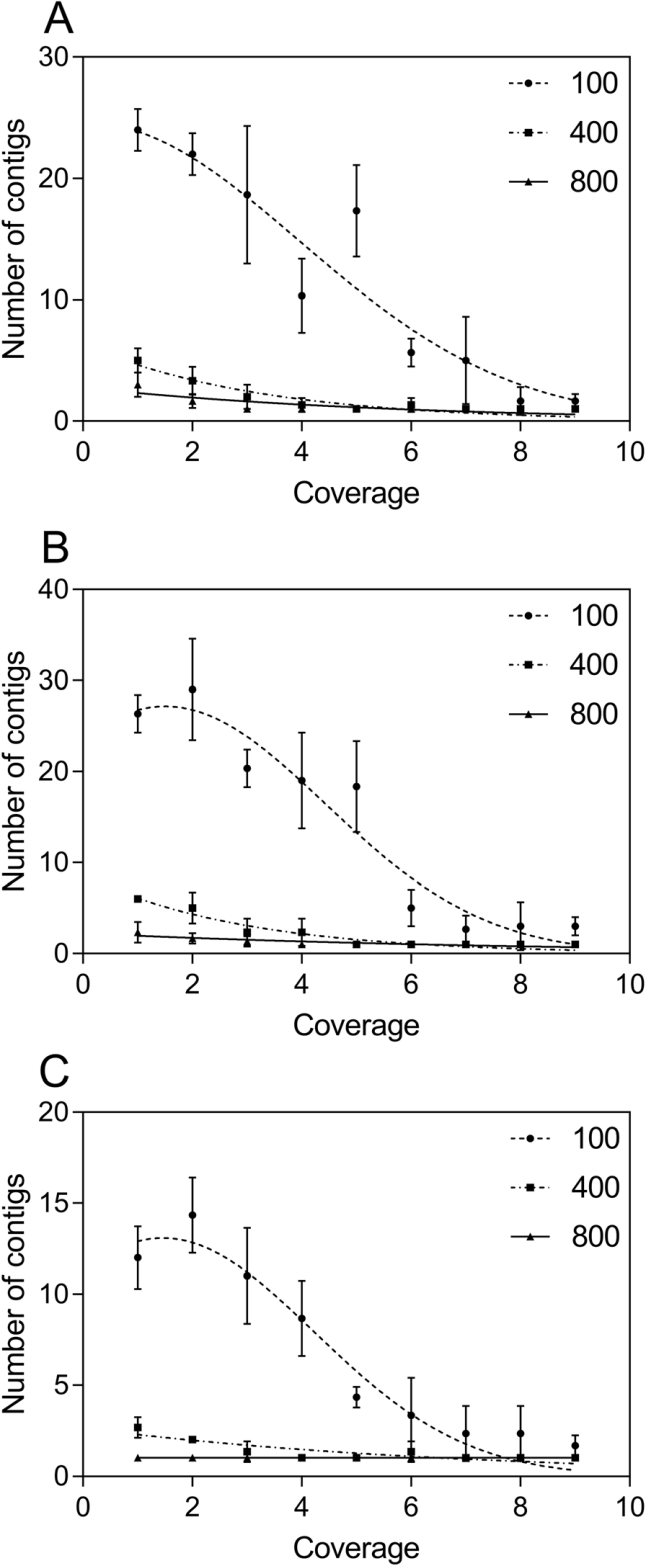
The relation between coverage, sequence length (nt), and the number of the assembled sequences (contigs) for the pCR™4-TOPO® (**a**), pQE-30-UA-mCHERRY-GFP (**b**), and pLEXSY-Ig-1 (**c**) cloning vectors using the Sequencer algorithm. The Gaussian distribution function was used for a curve fitting

**Fig. 3:**
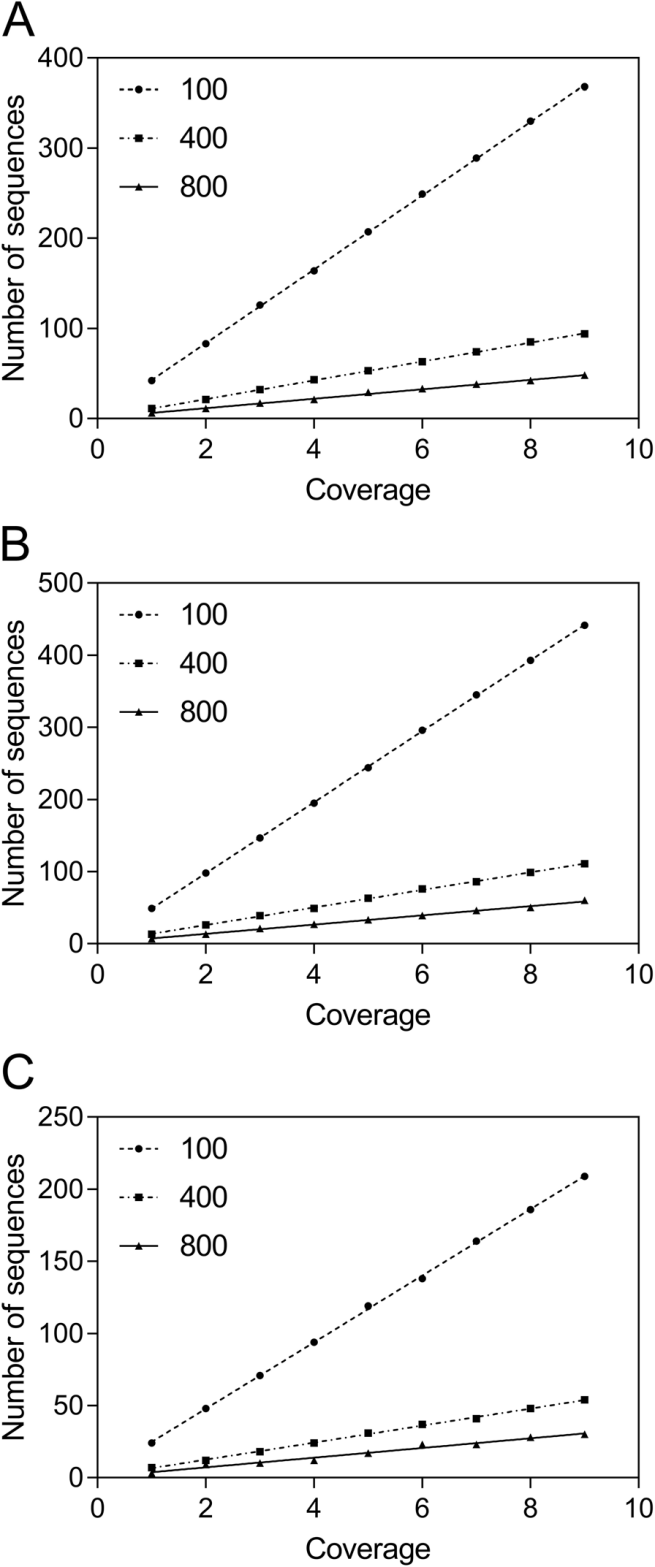
The relation between coverage, sequence length (nt), and the number of the generated sequences for the pCR™4-TOPO® (**a**), pQE-30-UA-mCHERRY-GFP (**b**), and pLEXSY-Ig-1 (**c**) cloning vectors using the Sequencer algorithm

**Fig. 4:**
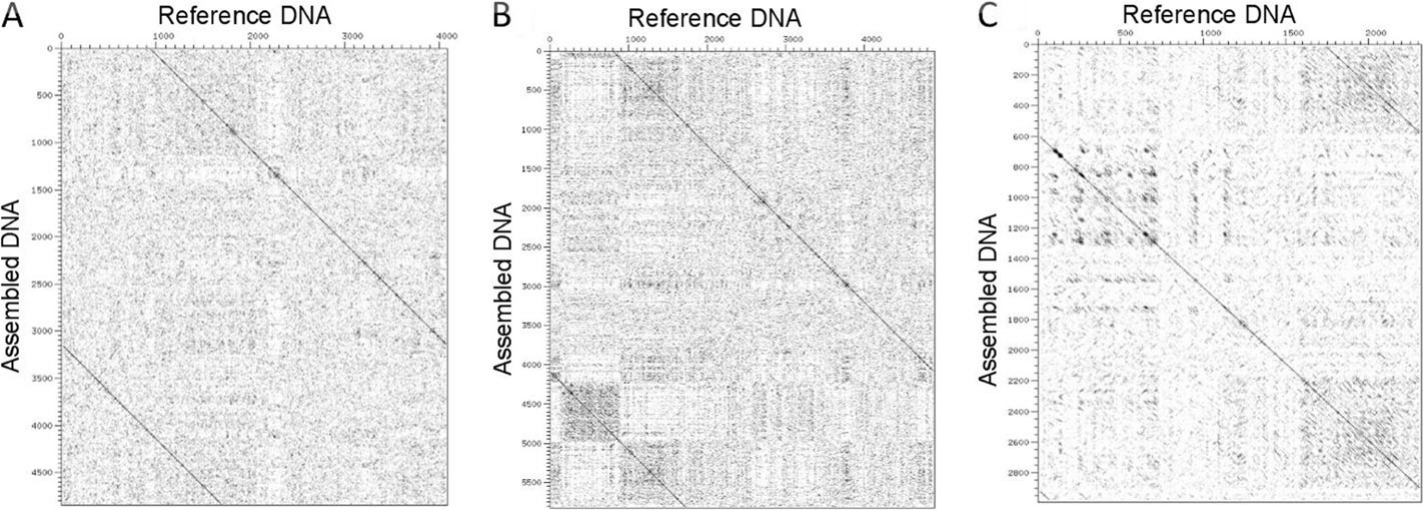
Dot plots of the pCR™4-TOPO® (**a**), pQE-30-UA-mCHERRY-GFP (**b**), and pLEXSY-Ig-1 (**c**) cloning vectors (*x*-axis, nt) versus the assembled DNA molecules with the maximal sequence length and coverage (*y*-axis, nt) using the Sequencer and TIGR Assembler algorithms

Next, we implemented the TIGR Assembler algorithm in order to recover the original cloning vectors and calculate the number of the assembled sequence fragments as contiguous sequences. Optimally, the program was designed to generate a single contiguous string from the various overlapping DNA fragments [16]. In order to test the quality of sequencing, we used different sequence lengths and coverage parameters to produce one contiguous sequence, which was then observed for all the analyzed vectors (Fig. 2 [a-c]), starting from the coverage number of 4 and higher sequence lengths (400 nt). Notably, the number of contigs at low sequence length (100 nt) fits a Gaussian distribution with reliable statistics (*r*^2^ = 0.83–0.86), whereas this parameter is linearly distributed at higher sequence lengths. Similar patterns had been previously inspected as multivariate distributions of tetranucleotide frequencies of artificial DNA fragments, where these distributions can be approximated by a single Gaussian function [26]. On the other hand, the results corresponding to 800 nt also correlate with the number of sequences for all the analyzed plasmids (Fig. 3 [a-c]) produced by the Sequencer algorithm, which is minimal at maximal sequence length. Consequently, the relationship between coverage and the number of sequences for the analyzed cloning vectors was estimated by the linear regression analysis with reliable statistics (*r*^2^ = 0.99) and a *p*-value of < 0.0001. The diagonal lines in Fig. 4 [a-c], indicating the DNA molecules generated and assembled by the Sequencer and TIGR Assembler algorithm, are corresponded to the template DNA as negative slopes (m < 0) of the lines from the reverse DNA strand. Furthermore, the pairwise sequence alignments using the CLUSTALW and BioEdit programs at the maximal value of sequence length and coverage indicated the exact match between the reference cloning vectors and the assembled DNA. However, the virtual DNA fragments were assembled in the manner (“impaired topology”), where the uncovered DNA located at the beginning of the reference DNA was complementary to the uncovered DNA located at the end of the assembled sequence (Fig. 4). This “impaired topology” outcome might be associated with the development of TIGR Assembler based on the data derived from more than 20 sequencing projects, leading, however, to a sequence assembler that produces some misassemblies [27]. Despite this drawback, the algorithm has been successfully implemented in whole-genome shotgun sequencing of prokaryotic and eukaryotic organisms, bacterial artificial chromosome-based sequencing of eukaryotic organisms, and expressed sequence tag assembly [16, 28, 29].

To access the local sequence alignment, the expectation value (*E*-value) as the expected number of local alignments with a given score (*S*) was calculated according to the Karlin-Altschul statistics (Table 2), using the following equation [18]:

$$ E=K\ast mn{e}^{-\lambda S} $$

where *m* is the MSP sequence length; *n* is the size of the database (*n* = 1 as no database was used); *e* is the exponential function; and *K* and *λ* are the search-space related and normalizing constants, respectively.

**Table 2:** Karlin-Altschul statistics for the analyzed DNA sequences

Vector	K	λ	MSP score	MSP length (nt)	Number of dots (*10^6^)
TOPO^*^	0.17	0.19	39.03	21	39.48
QE^**^	0.17	0.19	39.06	21	56.85
LEXSY^***^	0.16	0.18	41.55	24	13.85

^*^-pCR™4-TOPO®; ^**^- pQE-30-UA-mCHERRY-GFP; ^***^- pLEXSY-Ig-1; MSP-maximal-scoring segment pairs

**Fig. 5:**
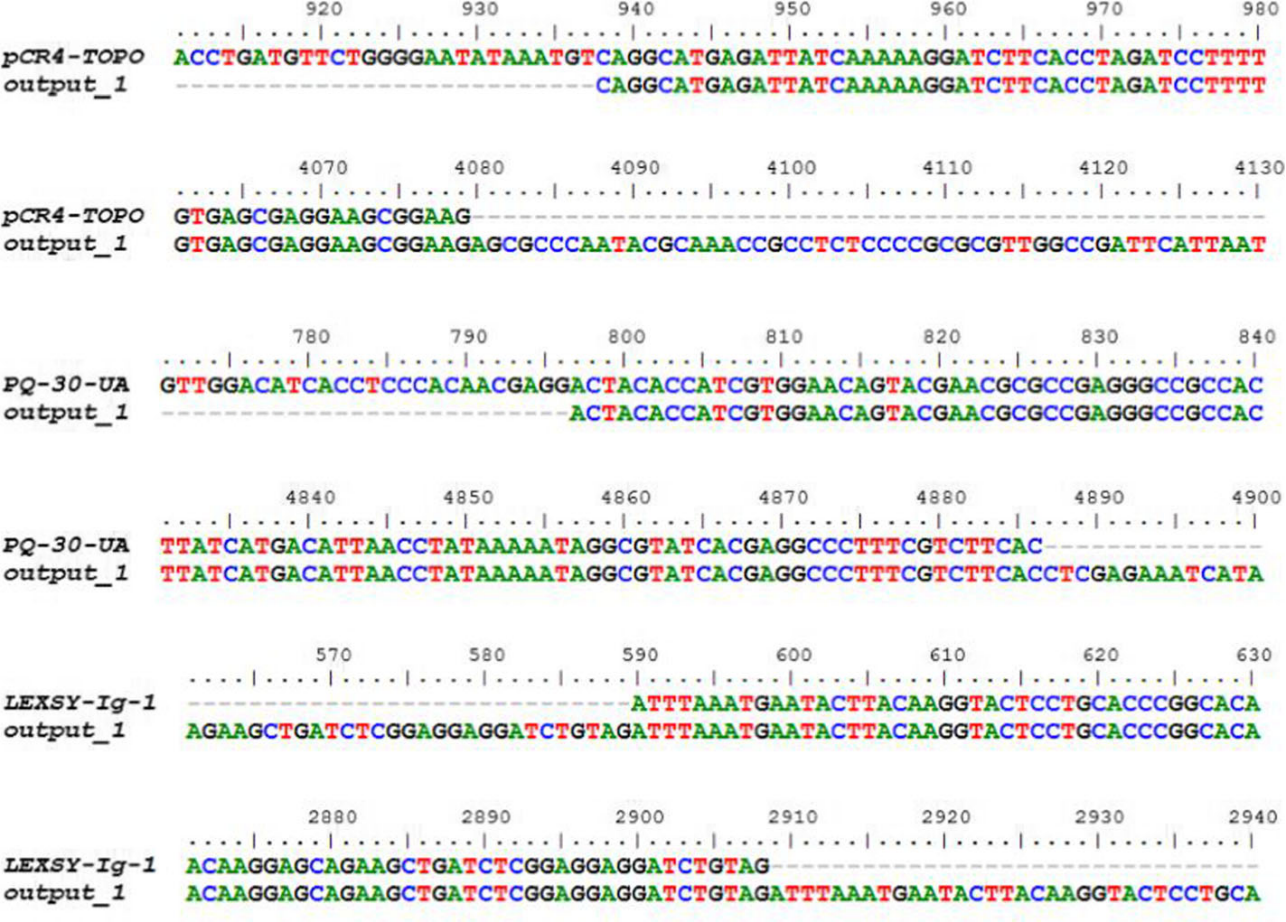
DNA sequence alignments of the analyzed cloning vectors as reference versus the assembled DNA molecules (output_1) produced by the Sequencer algorithm at the maximal value of sequence length and coverage, using the CLUSTALW and BioEdit programs. The starting and ending parts of the sequences are shown to enhance overall clarity

For the local alignments, the *E*-values were found to be the same (0.002) for all the analyzed vectors, indicating the statistically significant (*E*-value < 0.05) data produced by the Karlin-Altschul approach. Nonetheless, it has been shown previously that *E*-values might be dependent on the query sequence length, which might generate some “false positive” hits, previously observed, analyzing short primer regions and small domain regions [30, 31]. On the other hand, the DNA composition and identity analysis (Table 3) for the reference and assembled DNA revealed that their GC and AT contents were almost identical at the minimum vector size (pLEXSY-Ig-1) and the highest identity value (0.78). From the previous DNA sequence alignments (Fig. 5), it is clear that the identity values depend on the vector size and the “impaired topology” of the assembled DNA generated by the virtual sequencing algorithm. It has also been reported for NGS that extreme base compositions could lead to uneven coverage of reads, hindering genome assembly [32]. However, our experiments were conducted using the balanced GC and AT biases (~ 40–60%), which prevents the results from sequencing errors related to GC-poor sequences with a mean GC content of less than 25% [33].

**Table 3:** DNA composition and identity analysis for the reference (Ref) and assembled (Ass) DNA molecules

Parameter	DNA					
	TOPO^**^		QE^**^		LEXSY^***^	
	Ref	Ass	Ref	Ass	Ref	Ass
GC, %	51.7	52.44	48.32	46.96	58.3	58.51
AT, %	48.3	47.56	51.68	53.04	41.7	41.49
MW, kDa	1239.67	1470.74	1485.06	1767.37	710.69	915.77
Identity	0.54		0.62		0.78	

^*^-pCR™4-TOPO®; ^**^- pQE-30-UA-mCHERRY-GFP; ^***^- pLEXSY-Ig-1; MW- molecular weight calculated for a single stranded DNA

To validate the in silico output, the PCR methodology was applied to calculate the number of DNA copies (*n*) produced by amplifying the different sequence lengths (100, 400, and 800 nt) of the analyzed vectors (Additional file 2), using the following equation [34]:

$$ n=\frac{\left(a\times {N}_A\right)}{l\times 650\times {10}^9} $$

where *a* is the amount of DNA; *N*_*A*_ is the Avogadro constant; *l* is the sequence length (nt). The PCR technique largely depends on several factors, such as a polymerase, number of cycles, probe degradation, template volume and size of the reaction mixture [35, 36]. In contrast, virtual sequencing is a fully independent system, mimicking the sequencing strategy and identifying novel features of genomes, namely the satellite repeats, variations, and single-nucleotide polymorphism. Whereas our primers designed to amplify the specific DNA parts, the virtual sequencing allows targeting the random areas, which enables it to be used for de novo sequencing of random DNA fragments. For the experimental generation of truly novel plasmids in their native host, where the genetic material requires a correct assembly, it might be necessary to enrich and purify the plasmid DNA [37]. This can be achieved by closing the sequence gaps between contigs by PCR-amplification and subsequent Sanger sequencing of the PCR-product [37]. In our case, to provide consistent results, we used the same conditions for each experiment, including the design of primers with the same melting temperature, reaction time, and amount of reaction mixture. In addition, we used the fixed number of DNA copies (10 ng), contributing to the sequencing accuracy in our experiments.

**Fig. 6:**
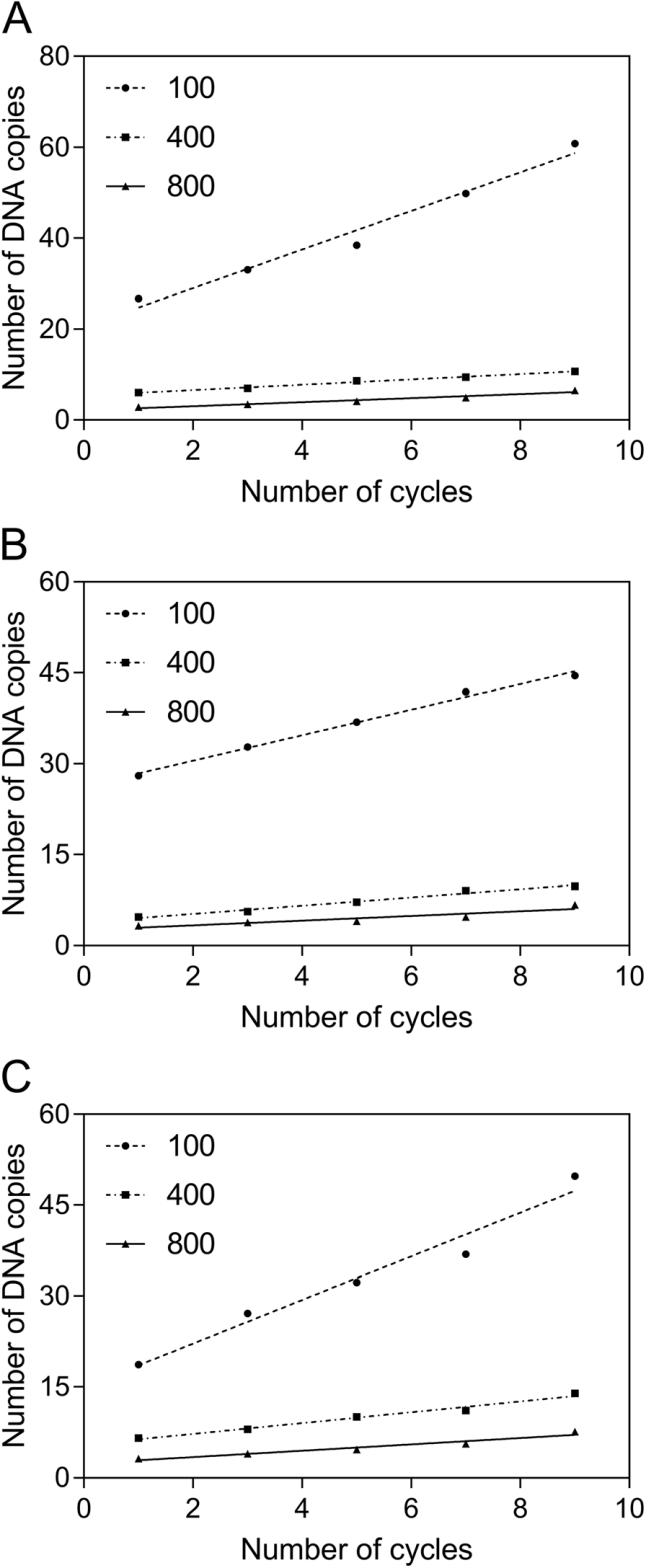
The relationship between sequence length (nt), the number of DNA copies (× 10^11^), and PCR cycles for the pCR™4-TOPO® (**a**), pQE-30-UA-mCHERRY-GFP (**b**), and pLEXSY-Ig-1 (**c**) cloning vectors using PCR

As an outcome, the numerical and experimental data corroborated reasonably for the number of DNA copies, which was minimal at 800 nt of sequence length, representing a relationship with average *r*^2^ and *p*-values of 0.92 and 0.012 for all the analyzed genetic elements (Fig. 6 [a-c]). This demonstrates that the number of amplicons in the PCR experiments and virtual sequencing correlate with the numbers of cycles of coverage rate consistently. Therefore, this technique can be applied (i) as an inexpensive quality control technique for sequencing analysis and (ii) as a support for the user with a reduced sequencing budget to emphasize sequencing data in silico. Currently, several sequencing strategies are available to determine the correct DNA sequence [38, 39]. Further applications of in silico sequencing algorithms might include the single-molecule sequencing method, able to analyze short-length segments in a large volume, which does not require the amplification of a DNA template [40] together with old-fashioned but very precise methods, such as the Sanger sequencing [41, 42].

## Conclusion

We tested and validated a novel virtual sequencing algorithm able to simulate shotgun sequencing. In reality, these simulations are challenging and require the implementation of multi-step protocols, including data production, assembly, and validation. Despite all the recent sequencing improvements, the Sanger method is still considered the “gold standard” in DNA sequencing due to its high accuracy. Therefore, in this study, we applied a novel algorithm to simulate shotgun sequencing and to build all possible consensus sequences from small DNA fragments for the gene-expression vectors. Therefore, the virtual sequencing approach was validated experimentally using the PCR technique with the number of cycles from 1 to 9 for each reaction. Overall, the obtained results can be used to correctly predict and emphasize the performance of this DNA sequencing technique based on the average sequence length to adjust the coverage values in experimental settings.

## Supplementary information

**Additional file 1.** Input and output data for modeling of shotgun sequencing of DNA plasmids. All the necessary files (tested in Linux environment) required for the virtual sequencing, including executable programs, bash scripts, FASTA format files, and program outputs.

**Additional file 2.** PCR results for plasmid vectors. To validate the in silico of DNA plasmids, the PCR methodology was applied to calculate the number of DNA copies produced by amplifying the different lengths (100, 400, and 800 nt) sequences of the plasmid vectors.

## Data Availability

Project name: Virtual sequencing project. Project home pages: https://github.com/virtualscreenlab/Virtual-Screen-Lab/blob/master/Virtual%20sequencing%20project.zip Operating system(s): Linux. Programming language: C. Other requirements: gcc, gawk, ranlib. License: GNU GPL. Any restrictions to use by non-academics: none.

## Abbreviations

NGS: Next-generation sequencing
MSP: Maximal-scoring segment pairs
GC: Guanine-cytosine content
AT: Adenine-thymine content
PCR: Polymerase chain reaction
DNA: Deoxyribonucleic acid
TIGR: The Institute for Genomic Research
